# Inhibition of ethylene involved in resistance to *E. turcicum* in an exotic-derived double haploid maize population

**DOI:** 10.3389/fpls.2023.1272951

**Published:** 2023-10-06

**Authors:** Sarah Lipps, Alexander E. Lipka, Santiago Mideros, Tiffany Jamann

**Affiliations:** Department of Crop Sciences, University of Illinois, Urbana, IL, United States

**Keywords:** *Exserohilum turcicum*, maize, BGEM, ethylene, disease resistance, quantitative disease resistance, GWAS

## Abstract

Northern corn leaf blight (NCLB) is an economically important disease of maize. While the genetic architecture of NCLB has been well characterized, the pathogen is known to overcome currently deployed resistance genes, and the role of hormones in resistance to NCLB is an area of active research. The objectives of the study were (i) to identify significant markers associated with resistance to NCLB, (ii) to identify metabolic pathways associated with NCLB resistance, and (iii) to examine role of ethylene in resistance to NCLB. We screened 252 lines from the exotic-derived double haploid BGEM maize population for resistance to NCLB in both field and greenhouse environments. We used a genome wide association study (GWAS) and stepwise regression to identify four markers associated with resistance, followed by a pathway association study tool (PAST) to identify important metabolic pathways associated with disease severity and incubation period. The ethylene synthesis pathway was significant for disease severity and incubation period. We conducted a greenhouse assay in which we inhibited ethylene to examine the role of ethylene in resistance to NCLB. We observed a significant increase in incubation period and a significant decrease in disease severity between plants treated with the ethylene inhibitor and mock-treated plants. Our study confirms the potential of the BGEM population as a source of novel alleles for resistance. We also confirm the role of ethylene in resistance to NCLB and contribute to the growing body of literature on ethylene and disease resistance in monocots.

## 1 Introduction

Northern corn leaf blight (NCLB), caused by the fungus *Exserohilum turcicum* (Pass.) K. J. Leonard and Suggs [syn*. Setosphaeria turcica* (Luttr.) K. J. Leonard and Suggs.], is one of the most important diseases of maize. *E. turcicum* is well-adapted to most maize growing regions in the world and causes yield losses globally (Savary et al., 2019). Over 7.62 million metric tons (300 million bushels) have been lost due to NCLB between 2016 and 2019 in the United States and Ontario, Canada (Mueller et al., 2020). The cost of economic losses due to damage from NCLB was estimated in one study examining hybrids with a range of resistance levels to be from $122.00 ha^-1^ to $353.20 ha^-1^ in fields managed for optimized yield (De Rossi et al., 2022). Furthermore, *E. turcicum* has a high evolutionary potential, as the fungus has high genetic variability and undergoes sexual reproduction in the field. Thus, there is the potential for widespread loss of resistance to *E. turcicum* (McDonald and Linde, 2002; Galiano-Carneiro and Miedaner, 2017; Munoz-Zavala et al., 2023).

The disease cycle and pathogenesis process of *E. turcicum* have been well characterized (Kotze et al., 2019; Navarro et al., 2020). *E. turcicum* is a hemibiotroph with a biotrophic and a necrotrophic phase. The fungus overwinters as mycelia in crop residue. Conidia are spread to the host by wind and rain. In the biotrophic phase, an appressorium-like structure is formed, and the fungus penetrates the cuticle, grows through the mesophyll, then colonizes the xylem (Navarro et al., 2020). The necrotrophic phase of the disease cycle begins when the fungus exits the xylem and colonizes the mesophyll, resulting in cell death. Eventually, *E. turcicum* forms conidiophores resulting in polycyclic disease over the course of the growing season (Kotze et al., 2019; Navarro et al., 2020). The first symptoms of infection are tan flecks that develop into tan-grey ovular lesions. The most severe epiphytotics are observed when disease symptoms occur before flowering time and in warm, humid environments (Ullstrup and Miles, 1957; Raymundo and Hooker, 1981a; Perkins and Pedersen, 1987).

An integrated NCLB management program includes host resistance, fungicides, and cultural methods. Host resistance is an important management technique as it does not increase the cost of production and does not harm the environment (Galiano-Carneiro and Miedaner, 2017). Both qualitative resistance, conferred by a single gene, and quantitative resistance, conferred by multiple genes, have been characterized in maize. Qualitative resistance genes effective for managing *E. turcicum* include *Ht1, Ht2, Ht3*, and *HtN1* (Welz and Geiger, 2000; Hurni et al., 2015; Galiano-Carneiro and Miedaner, 2017). *Ht2, Ht3*, and *HtN1* are allelic and encode a wall-associated protein kinase (Hurni et al., 2015; Yang et al., 2021). *Ht1* encodes a nucleotide-binding leucine-rich repeat receptor, *PH4GP- Ht1* (Thatcher et al., 2022). In this pathosystem, the qualitative genes do not confer complete resistance, but instead delay the onset of symptoms after initial infection and or dramatically reduce the severity of symptoms.

Many quantitative trait loci (QTL) effective against NCLB have been identified in maize (Wisser et al., 2006). A recent meta-analysis evaluating 110 studies identified chromosomes 10, 6, 5, 1, and 2 as having the highest odds of contributing a major-effect QTL for resistance to fungal and viral diseases, with chromosome 10 have the highest likelihood and chromosome 2 having the lowest likelihood (Rossi et al., 2019). In addition to regions associated with resistance, progress has been made on identifying potential quantitative resistance mechanisms to *E. turcicum.* Receptor-like kinases (RLKs) have been implicated in resistance to NCLB and other diseases. Three FERONIA-like receptors (FLRs), a type of RLK, have been identified as conferring resistance to multiple fungal foliar diseases, including NCLB (Yu et al., 2022). Originally *pan1*, an RLK, was implicated in increasing susceptibility to *E. turcicum*, as well as *Pantoea stewartii*, in maize on bin 1.06 (Jamann et al., 2014). However, a recent study revealed that *pan1* is not responsible for the altered resistance but potentially an aldo-ketoreductase is responsible instead (Doblas-Ibáñez et al., 2019). Additionally, a remorin gene, *ZmREM6.3*, on bin 1.02 was implicated in resistance to *E. turcicum, P. stewartii* and *Puccinia sorghi* (Jamann et al., 2016). Recently, a transcription repressor, *ZmMM1*, was cloned in maize from teosinte, and found to confer a lesion mimic phenotype, as well as resistance to NCLB and other fungal diseases (Wang et al., 2021). Other biochemical processes, such as the production of phenylpropanoids, have also been implicated as mechanisms of resistance. In bin 9.02, a maize caffeoyl-CoA *O-*methyltransferase encoded by *ZmCCoAOMT2*, is involved in the production of phenylpropanoids and lignin and is associated with resistance to multiple diseases (Yang et al., 2017). The recent advances in understanding NCLB have elucidated both regions associated with and mechanism of resistance.

The role of hormones in pathogen defense has been reviewed (Glazebrook, 2005; Robert-Seilaniantz et al., 2011; Denance et al., 2013). Three hormone pathways have been well discussed regarding their overlapping roles in abiotic and biotic stress: salicylic acid (SA), jasmonates (JA), and ethylene (ET). In Arabidopsis, SA is typically associated with biotrophic pathogens, and JA and ET are associated with necrotrophic pathogens (Glazebrook, 2005). Resistance to biotrophic and necrotrophic pathogens has been thought of as antagonistic, meaning that heightened resistance to biotrophic pathogens is typically associated with increased susceptibility to necrotrophic pathogens (Robert-Seilaniantz et al., 2011). However, the relationship between phytohormones and pathogen resistance is complex. Some fungi are able to mimic phytohormones or produce effectors that interfere with *in planta* hormone signaling (Denance et al., 2013). Additionally, pathogens are able to disrupt the balance of active hormones by targeting the regulatory aspects of hormonal pathways or inducing *in planta* hormone production (Robert-Seilaniantz et al., 2011).

The role of ethylene in disease resistance is complex. Ethylene is produced *in planta* in response to abiotic or biotic stress, which can be sensed by receptors located in the endoplasmic reticulum (Muller and Munne-Bosch, 2015). The ethylene pathway was first characterized in horticultural crops. It is synthesized from methionine which is converted into S-adenosyl-L-methionine (SAM) by SAM synthase. SAM is converted to 1-aminocyclopropane-1-carboxylate (ACC) via ACC synthase (ACS), and ACC is finally converted to ethylene via ACC oxidase (ACO) (Yang, 1985; Xu and Zhang, 2015). When activated, ethylene response factors link ethylene sensing and the activation of pathogen related genes (Huang et al., 2016).

The role of ethylene in plant-pathogen interactions has been extensively studied in dicots. However, several recent studies have focused on the role of ethylene in resistance against necrotrophic pathogens in maize. In maize, there are five known ACS genes and 13 ACO gene family members (Park et al., 2021). In kernels treated with *Fusarium verticillioides*, it was found that several ethylene production genes were induced upon infection, and ethylene production was associated with disease progression (Park et al., 2021). A similar trend was found in kernels inoculated with *Aspergillus flavus*, where ethylene production encouraged fungal conidiation and sporulation (Wang et al., 2017). Additionally, an ethylene signaling gene, *ZmEIN2*, regulated the abundance of metabolites associated with resistance to *Fusarium graminearum* in maize seedlings (Zhou et al., 2019). The bulk of research regarding ethylene in maize-pathogen interactions has focused on necrotrophic pathogens. Less is known about the role of hormone signaling and resistance to *E. turicucm*, a hemibiotrophic plant pathogen. Recently, an ethylene response factor, *ZmERF061*, was implicated in resistance against *E. turcicum* in maize (Zang et al., 2020; Zang et al., 2021). The plant’s ability to sense and respond to ethylene is likely important in defense against hemibiotrophic pathogens like *E. turcicum*, but this hypothesis needs further investigation.

Recent research shows that pathogens are increasing in complexity. In the case of *E. turcicum*, this increased complexity enables certain strains to overcome multiple, and occasionally, all available qualitative disease resistance genes (Weems and Bradley, 2018; Jindal et al., 2019; Muñoz-Zavala et al., 2022). The emergence of novel physiological races of *E. turcicum* requires further development and utilization of host resistance to effectively manage NCLB (Muñoz-Zavala et al., 2022). The endemic region of *E. turcicum* has expanded due to global warming; thus, it is likely that NCLB prevalence and severity are increasing in regions where it was previously a less common disease (Miedaner and Juroszek, 2021). With the increased complexity and ability of *E. turicucm* to overcome resistance in maize, it is imperative to continue identifying novel sources of resistance in maize. One effective approach to discover novel resistance alleles is by screening exotic-derived materials to identify previously unknown sources of resistance.

The germplasm enhancement of maize (GEM) program was established in 1995 to increase allelic diversity in temperate maize through the release of exotic-derived germplasm (Pollak and Salhuana, 2001). The BGEM population is an exotic-derived double haploid (DH) population developed by Iowa State University in collaboration with the GEM program (Brenner et al., 2012). In total, the BGEM population is derived from 67 landraces originating from 13 different countries (Sanchez et al., 2018). The BGEM population has previously been evaluated for flowering traits, kernel quality, and root architecture (Sanchez et al., 2018; Vanous et al., 2018; Vanous et al., 2019). We selected the BGEM population for this study as it is adapted to temperate climates and is a source of novel alleles not currently present in midwestern United States commercial germplasm.

The goals of this study were to (i) identify markers associated with resistance to NCLB in the BGEM population, (ii) identify potential metabolic pathways associated with resistance, and (iii) confirm the role of *in planta* ethylene production in NCLB resistance. We hypothesized that allelic diversity in the BGEM population would confer a range of resistance responses sufficient for genetic mapping when challenged with *E. turcicum.* Subsequently, we used the pathway association study tool (Thrash et al., 2020) to identify metabolic pathways associated with resistance. We confirmed the role of ethylene in the *E. turcicum-*maize pathosystem in the greenhouse by evaluating resistance responses of plants after treatment with AgNO_3_, a chemical which limits *in planta* ethylene action (Beyer, 1976; Kumar et al., 2009). Our results provide additional insight into the role of phytohormones and resistance to NCLB.

## 2 Materials and methods

### 2.1 Germplasm

The BGEM population is a BC_1_F_1_ exotic-derived DH population. The population was developed at Iowa State University in collaboration with the USDA-ARS Germplasm Enhancement of Maize project (Pollak and Salhuana, 2001). Briefly, BC_1_F_1_ lines were created by crossing exotic accessions to one of two expired Plant Variety Protection (PVP) lines, namely PHZ51 or PHB47, backcrossing the resulting F_1_ accessions were backcrossed to their respective ex-PVP parent, and then DH lines were created and self-pollinated (Brenner et al., 2012). Any lines that were infertile, lacked uniformity, or with poor agronomic traits were discarded. Genetically, the BGEM population is comprised of roughly 25% donor parent and 75% recurrent parent (Sanchez et al., 2018). The BGEM population represents a broad diversity of exotic derived maize. In total 67 landraces from 13 different countries are represented (Sanchez et al., 2018).

### 2.2 Experimental design

The BGEM population was screened for resistance to *E. turcicum* in three environments from 2019 to 2021. In total, all lines were evaluated four times in the field between 2019 and 2021 and twice in the greenhouse in 2020. All experiments were designed as augmented randomized incomplete block designs using the *agricolae* package (de Mendiburu, 2021) in the statistical software R, version 3.6.0 (R Core Team, 2021). Lines from BGEM population were screened at the Crop Sciences Research and Education Centers in Urbana, IL in 2019 (n = 252) and 2021 (n = 240). Differences in the number of lines used in 2019 and the other years were due to seed availability. Field plots were planted in rows that were 3.65 m long with 0.91 m alleys. The 2019 field was irrigated to encourage disease development. Lines were replicated twice in 2019 and 2021. In 2019, incomplete blocks (n = 14) were augmented with Oh7B and NC344 as susceptible and resistant checks, respectively. In 2021, Oh7B, NC344, PHB47, and PHZ51 served as check lines in each incomplete block (n = 16). In 2021 the following NCLB differential lines were included in each replication: A619-*Ht1*, A619-*Ht2*, A619-*Ht3*, A619, B37-*HtN1*, and B37.

In 2020, BGEM lines (n=240) were screened at the Plant Care Facility in Urbana, IL. The 2020 greenhouse experiment was replicated twice with 13 incomplete blocks in each replication. Due to space constraints, replications had to be run separately. One plant per line was grown in a one-gallon pot filled with 1:1:1 general purpose potting mix. Greenhouse conditions were set to 12/12 hr. light-dark cycle. Ambient temperatures were set to 24-28°C during the day and 20-22°C during the night. Lines were organized in an augmented randomized incomplete block design. Oh7B and NC344 were susceptible and resistant check lines in each block. PHB47 and PHZ51 were randomized once in each replication. The same qualitative check lines used in 2021 were also used in the greenhouse in each replication.

### 2.3 Inoculation and phenotyping

In 2019 natural inoculum was relied upon for infection, as the growing season was conducive for disease development and evaluation. For all other experiments, inoculum was prepared using five fungal isolates (19StM06, 19StM07, 19StM09, 19StM11, 19StM12) that were isolated from diseased maize tissue collected from the 2019 field experiment at Crop Science Research and Education Center in Urbana, IL. A mixture of isolates was used to best reproduce disease pressure observed in 2019. For the 2020 greenhouse experiment, plants were inoculated with a spore suspension at the V3 stage (Adhikari et al., 2021). Fungal isolates were grown on lactose-casein hydrolysate agar (LCA) media for 14 days under 12/12 hr light/dark photoperiod at room temperature. Spores were harvested from 14-day old plates and the concentration adjusted to 4 × 10^3^ spores/mL in a 0.02% Tween 20 solution. For inoculations, 0.5 mL of the spore suspension was pipetted into the whorl of the plants. Following inoculation, plants were maintained in high humidity conditions for 24 hours to facilitate disease development. In 2021 plants were inoculated at the V4 to V5 stage using infested sorghum grains (Zhang et al., 2020). Fungal isolates were cultured on LCA for 14 days as described above. After 14 days of culturing, agar pieces cut directly from the plates were added to mushroom bags containing 1000 mL of soaked, autoclaved sorghum grains. The mushroom bags were cultured for two to three weeks at room temperature under 12/12 hr light/dark photoperiod. The infested grains were examined under a dissecting microscope to confirm the presence of conidia then dried and stored at room temperature. Each plant was inoculated with 1.3 g of the prepared inoculum in the whorl at the V3 growth stage.

Disease ratings for the 2019 and 2021 field experiments were taken on a whole plot basis using a 0-100% scale in 5% increments (Poland and Nelson, 2011) where no disease present was represented by 0%, and a rating of 100% indicated that the total leaf area of the plants was necrotic due to disease. In 2019 two field ratings were taken after lines had started flowering, and ratings were taken 7 days apart. In 2021 a total of four field ratings were taken. Ratings were conducted every 7-14 days starting two weeks before the onset of flowering in the earliest maturing plants.

Lines in the 2020 greenhouse experiment were evaluated for incubation period and disease severity. Incubation period is defined as the number of days after inoculation (DAI) that the first lesions were visible. Lines were evaluated for incubation period every 48 hours until all plants showed lesions. In the greenhouse experiment, a total of four diseased leaf area ratings were taken. Disease leaf area ratings were taken every 7-14 days starting the day when all plants had lesions present. Lines were rated on a single leaf per plant basis using the same 0-100% rating scale mentioned above.

### 2.4 Statistical analyses

Disease severity data was examined using the standardized area under the disease progress curve (sAUDPC). sAUDPC is used to compare disease progression between environments or experiments (Simko and Piepho, 2012). sAUDPC is calculated by first measuring the area under the disease progress curve (AUDPC), which is the area of a trapezoid between two or more time points on a progression curve and accounts for disease severity over time (Jeger and Viljanen-Rollinson, 2001; Madden et al., 2007). The sAUDPC value is then obtained by dividing AUDPC by the weighted total of the number of days of disease evaluation. sAUDPC was calculated using the R package *agricolae* (de Mendiburu, 2021). Both days to anthesis (DTA) and days to silking (DTS) were recorded for the 2019 field experiment. DTA is defined as the number of days after planting when 50% or more of the plot had visible anthers on the tassel. DTS is defined as the number of days after planting when 50% or more of the plot had visible silks emerging from the ears. Pearson correlation coefficients for sAUDPC across all environments were calculated using the *rcorr()* function in the R package *Hmisc* v4.5-0 (Harrell, 2021). Flowering data was only collected in 2019. Using AUDPC, DTS, and DTA data from 2019, Pearson correlation coefficients were calculated between each trait.

Both sAUDPC and incubation period values were used to fit mixed models and estimate least squared means (LS Means). The following model was fit to estimate LS Means for sAUDPC in the 2019 + 2021 dataset:


$${\text{Y}}_{\text{ijkl}}=\;µ+\;{\;}_{\text{Gi}}+\;{\;}_{\text{Rj}}+\;{\text{B}}_{\text{l}\left(\text{j}\left(\text{k}\right)\right)}+\;{\text{E}}_{\text{l}}+\text{G}{\text{E}}_{\text{il}}+{\text{ε}}_{\text{ijkl}}$$

The factors are defined as follows: *Y_ijkl_* is the sAUDPC value from genotype *i* in replicate *j* in block *k* and environment *l*; *G_i_* is the fixed effect of genotype *I*; *R_j_* is the random effect of replicate *j*; *B_k(j)_* is the random effect of block *k* nested in replicate *j* nested in environment *l*; *E_l_* the random effect of the environment *l; GE_il_* the random effect of the interaction between genotype *i* and environment *l*; and *ε_ijkl_* the error term. From this model, LS Means were calculated from the estimates of G_i_ from the fitted model. For the 2020 incubation period dataset the following mixed model was fit:


$${\text{Y}}_{\text{ij}}=\;µ+\;{\text{G}}_{\text{i}}+\;{\text{B}}_{\text{j}}+\;{\text{ε}}_{\text{ij}}$$

Terms in the model are described as follows *Y_ij_* is the incubation period value in days of genotype *i* in block *j*; *G_i_* is the fixed effect of genotype *i*; *B_j_* is the random effect of block *j*; and ε_ij_ the error term associated with *Y_ij_.* The fixed effects for genotype in each model were extracted and used for further analysis. As with the previous model, LS Means were calculated from the estimates of G_i_ from the fitted model.

### 2.5 Genotyping

The BGEM population was previously genotyped (Sanchez et al., 2018). A dataset consisting of 62,077 single nucleotide polymorphisms (SNPs) was obtained from Sanchez, Liu (Sanchez et al., 2018). This dataset was generated for the DH BGEM lines via genotyping-by-sequencing (Elshire et al., 2011) by the Cornell University Genomic Diversity Facility with data analysis by the Buckler Lab for Maize Genetics and Diversity. The 62,077 SNP dataset was generated by filtering SNPs with a large amounts of missing data and low allele frequencies, followed by SNPs in the same genetic position (Sanchez et al., 2018). Within the BGEM population, the average number of recombination events was higher than expected. A Bayes theorem described by Sanchez, Liu (Sanchez et al., 2018) was implemented to correct for monomorphic markers that were flanked by markers with donor parent genotypes. After correction, the number of recombination events was reduced, and the donor genome composition was closer to 25%, as expected. All edits to the genotypic dataset were conducted prior to this study.

### 2.6 Identification of markers associated with disease resistance

To identify candidate regions associated with resistance to *E. turcicum* two mapping strategies were employed using TASSEL v5.2.81 (Bradbury et al., 2007). First, we used a generalized linear model (GLM) in TASSEL to identify significant markers, as well as a stepwise regression approach to identify markers that were associated with disease severity and incubation period. The Bayesian corrected genotypic dataset was filtered to remove markers with a minor allele frequency less than 0.05. Two principal components, calculated in TASSEL using the standard PCA plugin, were used to control for population structure. The phenotypic datasets used were the LS Means estimated using the 2019 + 2021 combined disease severity and the 2020 incubation period datasets (**File S1**). The same genotypic dataset was used for both phenotypes. The GLM model was ran for both phenotypes with 1000 permutations. A significance threshold of α = 0.10 was applied using permutation corrected *p*-values. For the second approach, stepwise regression was implemented in TASSEL independently of the GLM association analysis. The two principal components calculated in TASSEL were included as numeric covariates along with the estimated LS Means value for each line. The stepwise regression analysis was implemented for both the 2019 + 2021 combined disease severity and the 2020 incubation period datasets. The *p*-value model was used with 1000 permutations with entry and exit limits of 1 × 10^-5^ and 2 × 10^-5^, respectively. A significance threshold of α = 0.10 was used to identify significant SNPs.

### 2.7 Pathways Association Study Tool

The Pathway Association Study Tool (PAST) approach (Thrash et al., 2020) was implemented via the MaizeGDB online platform (Woodhouse et al., 2021). PAST is designed to take outputs generated by TASSEL and provide biological insight into association study results. The software interprets the results from association analysis to identify metabolic pathways associated with genes that are strongly associated with the trait through significant markers or when genes are moderately associated with a trait, but the markers themselves may not have been significant in the original association study. PAST uses the output from association analysis, allelic effects files, and a linkage disequilibrium file to assign SNPs to genes based on LD and genomic distance between SNPs and genes before then identifying significant metabolic pathways. PAST analysis was conducted for the 2019 + 2021 combined disease severity dataset and the 2020 incubation period dataset. The association and effect files generated from the GLM were used as the input for the PAST analysis. A gene assignment window size of 1000 base pairs was used. Pathways with at least five genes were considered and significance was determined based on 1000 permutations. A Type I error rate of α = 0.05 was used to determine statistical significance for each pathway.

### 2.8 Differentially expressed genes associated with ethylene

A recent study examined differences in gene expression in maize and sorghum plants inoculated with different strains of *E. turicum* (Adhikari et al., 2021) (**File S2**). Our hypothesis was that genes involved in the synthesis of ethylene were differentially expressed in mock-inoculated maize plants versus *E. turcicum* inoculated maize plants. A list of genes associated with ethylene biosynthesis was downloaded from CornCyc7.5 available through the MaizeGDB (corncyc-b73-v3.maizegdb.org) (**File S3**).

Transcriptome data were generated by Adhikari et al. (2021). Briefly the maize line B73 was grown in the greenhouse and either inoculated with *E. turcicum* or mock inoculated with sterile deionized H_2_O (DI H_2_O). RNA was extracted from plant tissue collected at 24 and 72 hours after inoculation (hai) and sequenced at the Roy J. Carver Biotechnology Center at the University of Illinois at Urbana-Champaign. Quality control, alignment, and normalization were conducted using standard procedures (Adhikari et al., 2021). We used the final dataset with annotated differentially expressed genes (DEGs) and calculated false discovery rates (FDR) (Adhikari et al., 2021). (**File S2**). Global FDR adjusted p-values were calculated based on the Benjamini & Hochberg procedure (Benjamini and Hochberg, 1995).

Our objective with the RNA data was to examine the expression of genes associated with ethylene biosynthesis in maize during the *E. turcicum* infection stage. Therefore, we are interested in three contrasts: DEG of maize 24 and 72 hai with *E. turcicum*, DEG of maize 24 hai with *E. turcicum* and DI H_2_O, and DEG of maize 72 hai with *E. turcicum* and DI H_2_O. Using a list of known genes involved in ethylene biosynthesis (**File S3**), we filtered the normalized list of DEGs for those only involved in ethylene biosynthesis. Then, for each contrast we only examined DEGs with an FDR value greater than or equal to 0.05. We hypothesized that ethylene biosynthesis could be involved in maize early defense against *E. turcicum*.

### 2.9 Greenhouse ethylene assay

A subset of DH lines was chosen to evaluate the role of ethylene in resistance against *E. turcicum.* In maize, ethylene is inhibited by the foliar application of AgNO_3_ (Kumar et al., 2009). Using LS Means data from the 2021 field experiment, we examined disease phenotypes within exotic donor families. Only the ex-PVP recurrent parents have been genotyped, and there is no genotypic information for the landrace donor parents. We chose a resistant and susceptible line, as we wanted to see if inhibiting ethylene affected phenotypes of resistant or susceptible lines differently (**Table 1**). We selected pairs of lines categorized as resistant or susceptible within five exotic landrace groups. Both ex-PVP recurrent parents, PHB47 and PHZ51, were included in the assay. Our goal was to assess the role of ethylene between resistant and susceptible lines, within different exotic families, and within different recurrent parent backgrounds.

**Table 1:** Lines selected to evaluate the role of ethylene in resistance against *E. turcicum*.

Line	Landrace	RP^*^	Pedigree	R/S^^^
BGEM-0078-S	Cristalino Amarillo	PHB47	(CRISTALINO AMAR AR21004/PHB47 #001-(2n)-002	R
BGEM-0079-S	Cristalino Amarillo	PHB47	(CRISTALINO AMAR AR21004/PHB47 #005-(2n)-003	S
BGEM-0087-N	Dulcillo del Noroeste	PHZ51	(DULCILLO DE NO SON57/PHZ51)/PHZ51 #001-(2n)-001-001-B	R
BGEM-0088-N	Dulcillo del Noroeste	PHZ51	(DULCILLO DE NO SON57/PHZ51)/PHZ51 #002-(2n)-002	S
BGEM-0108-S	Elotes Occident	PHZ51	(ELOTES OCCIDENT NAY29/PHB47)/PHB47 #002-(2n)-001-001-B	R
BGEM-0109-N	Elotes Occident	PHZ51	(ELOTES OCCIDENT DGO236/PHZ51)/PHZ51 #004-(2n)-002-002-B	S
BGEM-0120-N	Jora	PHZ51	((Jora - ANC 1/PHZ61 B)/PHZ51)-(2n)-001-001-B	R
BGEM-0121-N	Jora	PHZ51	((Jora - ANC 1/PHZ61 B)/PHZ51)-(2n)-002-001-B	S
BGEM-0162-S	Morado	PHB47	(MORADO BOV567/PHB47)/PHB47 #002-(2n)-001	R
BGEM-0167-S	Morado	PHB47	(MORADO BOV567/PHB47)/PHB47 #005-(2n)-003	S
PHB47	-	-	-	R
PHZ51	-	-	-	R

^*^RP, recurrent parent.

^^^R/S, Resistant/Susceptible.

Lines were selected in pairs from five exotic landrace groups based on phenotype to see if exotic landrace background influenced ethylene production and if inhibiting ethylene influenced the phenotypes of resistant and susceptible lines differently. Both recurrent parents, PHZ51 and PHB47, were included in the experiment.

The experiment was arranged in a split-plot design. The whole-plot was a complete block of plants, and the subplot was each BGEM line randomly assigned to each treatment within each complete block. Lines were either treated with AgNO_3_ followed by inoculation with *E. turcicum*, or treated with DI H_2_O followed by inoculation with *E. turcicum*. There were three whole-plots (complete blocks) per treatment and each line occurred once in each whole-plot. Greenhouse growing conditions were the same as the conditions described previously. The *E. turcicum* spore suspension was prepared as explained above. At the V3 growth stage, plants were treated with either 20 mM AgNO_3_ in 0.001% Tween 20 (Wang et al., 2017) or with sterile water in 0.001% Tween 20 at a rate of 187 L ha^-1^ at 207 kPa using a spray chamber (Technical Machinery Inc., Sacramento, CA). The chamber was equipped with an even flat-fan nozzle 8002E (TeeJet Technologies, Wheaton, IL) and plants were sprayed 45 cm above the tallest leaf. After treatment, plants were allowed to dry completely before inoculation with 4 × 10^3^ spores/mL in a 0.02% Tween 20 solution prepared as mentioned previously. Plants were inoculated by pipetting 0.5 mL of spore suspension into the whorl. After inoculation, a high-humidity environment was maintained overnight to encourage disease development. All plants were evaluated for incubation period and disease severity on a per plant basis as described previously. AUDPC was calculated using the *agricolae* package (de Mendiburu, 2021) in the statistical software R, version 3.6.0 (R Core Team, 2021). We ran an ANOVA to appropriately account for the differences in the whole-plots and the subplot replicates.

## 3 Results

### 3.1 Characterization of the BGEM population

The BGEM population was evaluated for DTA and DTS in 2019 and for resistance to *E. turcicum* in 2019 and 2021 in the field at the Crop Sciences Research and Education Center in Urbana, IL. A field trial of the BGEM population was lost during the 2020 growing season due to poor emergence, followed by a hailstorm, which destroyed the field. Thus, the BGEM population was grown a second time and evaluated for resistance in the greenhouse in 2020 at the Plant Care Facility in Urbana, IL. All checklines performed as expected. In the 2019 and 2021 field environments, Oh7B was more susceptible than NC344. The BGEM recurrent parents, PHB47 and PHZ51, were moderately resistant in the 2021 field season with PHZ51 being the more resistant recurrent parent. The observed range of phenotypes suggest that there was sufficient disease pressure in each experiment to evaluate for disease severity and incubation period, even in the uninoculated field trial (**Table 2**).

**Table 2:** Variation for standardized AUDPC (sAUDPC), incubation period (IP), days to anthesis (DTA) and days to silk (DTS) in each environment the BGEM population was screened in.

Environment	Trait	Minimum	Maximum	Mean
2019 Field	sAUDPC^*^	17.50	87.50	42.59
2021 Field	sAUDPC^*^	8.07	49.43	27.62
2020 GH	IP^^^	5	19	10
2019 Field	DTA^#^	61	84	69
2019 Field	DTS^+^	63	84	71

^*^sAUDPC, standardized area under the disease progress curve.

^^^IP, incubation period, the number of days from inoculation to the appearance of first lesions.

#DTA, days to anthesis, the number of days after planting until 50% of the plot has visible anthers.

^+^DTS, days to silking, the number of days after planting until 50% of the plot has visible silks.

Due to the high evolutionary potential of *E. turcicum*, we examined whether isolates collected from Champaign County could overcome major genes associated with resistance. In 2020 and 2021 A619-*Ht1*, A619-*Ht2*, A619-*Ht3*, A619, B37-*HtN1*, and B37 were included to evaluate whether the mixture of 19StM06, 19StM07, 19StM09, 19StM11, 19StM12 were able to overcome major genes associated with resistance. In the 2020 greenhouse experiment we did not observe a significant difference in AUDPC or incubation period among lines containing major genes and their respective background based on a Dunnett’s test (α *=* 0.05), but we observed typical resistant responses for *Ht2, Ht3*, and *HtN1*. In the 2021 field experiment the AUDPC for A619-*Ht2* and A619-*Ht3* were significantly different from A619 based on a Dunnett’s test (*p<* 0.001), indicating that *Ht2* and *Ht3* conferred resistance. Additionally, B37-*HtN1* was significantly different from B37 (α = 0.01), indicating that *HtN* conferred resistance. Discrepancies between genotype performance in the greenhouse and the field environment could be due to other *E. turcicum* genotypes present in the field environment. The effectiveness of qualitative and quantitative under greenhouse conditions differs from the field environment (Raymundo and Hooker, 1981b; Chung et al., 2010). Thus, it is possible that the observed discrepancy in phenotypes between the field and greenhouse environments could be attributed to environmental effects on the plants, as well as additional strains present in the field environments.

**Figure 1:**
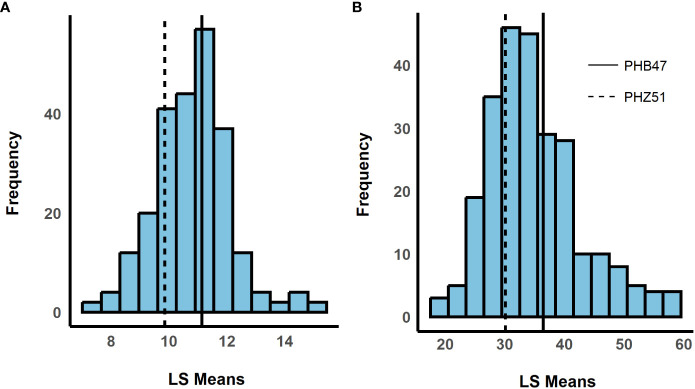
Histograms showing the distribution of least squared means (LS Means). The x-axis shows LS Means values, and the y-axis shows the frequency. LS Means were estimated for **(A)** incubation period and **(B)** standardized area under the disease progress curve (sAUDPC). Dashed and solid horizontal lines represent recurrent parents PHZ51 and PHB47, respectively. (180 x 100 mm, 300 dpi).

We observed a difference in resistance responses between the greenhouse and field environments. The 2019 and 2021 field environments were strongly and significantly correlated, (r= 0.64, α = 0.001) while the 2020 greenhouse environment had a weaker correlation with the 2019 field environment (r = -0.21, α = 0.01) and the 2021 field environment (r = -0.29, α = 0.01, **Table 3**). The strong correlation between the 2019 and 2021 field data underscores that similar levels of resistance were observed even though one field was artificially inoculated while the other relied on natural inoculum. In the mixed model for the 2019 + 2021 combined dataset, environment contributed the most variance, followed by the genotype × environment interaction (**Table 4**). The model used to estimate LS Means in the 2020 incubation period dataset only included block but not replication. Replication was not included as it had a covariance estimate of zero, indicating that block and replication were redundant. By including the blocking factor in the model, we are still able to account for variation in the greenhouse environment. The 2020 greenhouse diseased leaf area was analyzed in the same manner as the 2020 greenhouse incubation period. The estimated LS Means for the 2019 + 2021 combined dataset and the 2020 incubation period dataset showed a range of phenotypic responses suitable for association mapping (**Figure 1**, **Table 1**).

**Table 3:** Pearson correlation coefficients between LS Means disease severity among environments.

	2021 Field	2020 GH DLA^+^	2020 GH IP^^^
2019 Field	0.64^***^	0.16^**^	-0.21^**^
2021 Field		0.34^***^	-0.29^***^
2020 GH DLA			-0.37^***^

Significance ^***^ p ≤ 0.001, ^**^ p ≤ 0.01.

^+^DLA, diseased leaf area.

^^^IP, incubation period.

Field environments were evaluated for diseased leaf area (DLA) while greenhouse experiments were evaluated for incubation period (IP) and diseased leaf area.

**Table 4:** Covariance estimates with standard deviation for all fixed factors included in each mixed model used to estimate LS Means for incubation period and the 2019 + 2021 combined disease severity datasets.

Factor	2019 + 2021 Fields	Incubation Period	2020 GH DLA
Environment	95.65(9.78)	−	−
Genotype × Environment	17.20(4.15)	−	−
Environment/Replication/Block	11.71(3.42)	−	−
Environment/Replication	3.62(1.90)	−	−
Block	−	0.17 (0.41)	5520 (74.29)
Residuals	31.75(5.6)	2.32 (1.52)	5499 (74.16)

### 3.2 Identification of significant markers associated with resistance to NCLB

**Figure 2:**
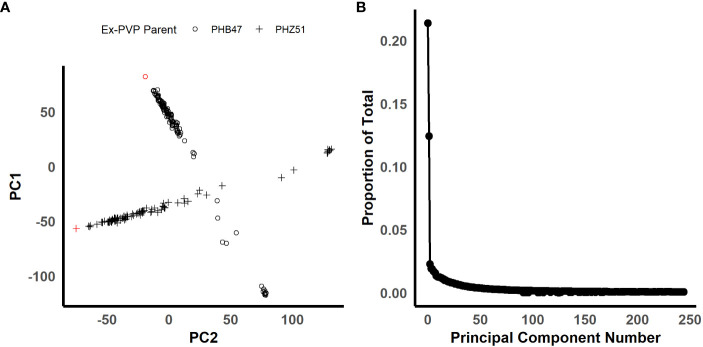
Figures illustrating the population structure of the BGEM population. **(A)** Is a scatter plot for principal components 1 and 2 plotted against each other. The shape of the points represents each ex-PVP background. The ex-PVP parents, PHB47 and PHZ51, are highlighted in red. **(B)** Is a scree plot of all principal components. The x-axis is the principal component number, and the y-axis is the proportion of total variation explained by each principal component. (200 x 100 mm, 300 dpi).

**Figure 3:**
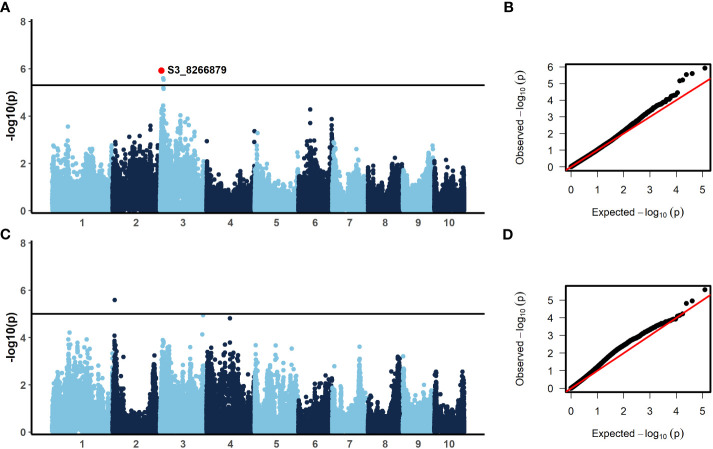
Results from the generalized linear model (GLM) with two principal components to control for population structure **(A, C)** and qq-plots showing observed versus expected -log_10_(*p*) **(B, D)**. A significance threshold of α=0.1 was established based on 1000 permutations conducted in TASSEL. For incubation period, evaluated in the greenhouse in 2020, **(A, B)** three SNPs were significant. The annotated SNP, S3_8266879, was also significant with a stepwise regression model. For the 2019 + 2021 combined field sAUDPC data, **(C, D)** one SNP was significant. (300 x 180 mm, 300 dpi).

In order to identify candidate markers associated with resistance to NCLB, we employed genome-wide association analysis using TASSEL (Bradbury et al., 2007). To control for population structure, two principal components were included in the model, as most of the variation is explained by the first two components (**Figure 2**). We then employed a GLM model using TASSEL to examine the genetic architecture of resistance to NCLB. For both the 2019 + 2021 field dataset and the 2020 greenhouse incubation period dataset log quantile-quantile plots were constructed to assess the effectiveness of two principal components to control population structure (**Figures 3B, D**). Given the genetic design of the BGEM population, this approach is appropriate to control for population structure while controlling for any false positives. The same mapping approach was used for the 2019 + 2021 field dataset, the 2020 greenhouse incubation period dataset, and the 2020 greenhouse diseased leaf area dataset. We did not identify any significant markers using the 2020 greenhouse diseased leaf area dataset. In total, four significant markers were identified using the GLM model in TASSEL (**Figure 3**).

The BGEM-DH lines have a high percentage of recurrent parent genome, and as such, linkage disequilibrium (LD) decays at a much slower rate than expected. In some regions, the LD blocks are large and span 100,000,000 bp or beyond (Sanchez et al., 2018). Given the LD structure in this population, we considered genes within 1 Mbp of a significant marker when examining candidate genes. Three markers on chromosome 3 were associated with delayed incubation period (**Table 5**, **Figure 3A**). A stepwise regression approach using the same marker dataset identified a single significant marker for incubation period on chromosome three (S3_8266879; *p =* 5 × 10^-5^), which was also significant in the GLM model (*p =* 0.033, **Table 5**, **Figure 3A**). The marker S3_8266879 is within GRMZ2G108898, plant cysteine oxidase 1 (*PCO1*). *PCO1* spans from 8,263,929 bp to 8,269,675 bp on chromosome 3. The other two significant markers identified in the GLM model for incubation period, S3_15712704 (*p =* 0.53) and S3_18008517 (*p =* 0.55) were proximal to GRMZM2G445261 a probable carboxylesterase 15 and GRMZM2G380195 a pentatricopeptide repeat-containing protein chloroplastic, respectively. While these genes were the closest to the most significant marker, it is important to keep in mind the extensive LD structure in this population.

**Table 5:** SNPs associated with resistance to *E. turcicum* were identified in the BGEM populations using genome-wide association and stepwise regression models.

Dataset	Chr.^*^	SNP^^^	Perm. *p*-value (GWAS)	*p*-value (Step Reg.)	Nearest gene	Distance from marker (BP)
Incubation Period	3	S3_8266879	0.033	5 × 10^-5^	GRMZM2G108898	Genic
Incubation Period	3	S3_15712704	0.053	−	GRMZM2G445261	10,690
Incubation Period	3	S3_18008517	0.055	−	GRMZM2G380195	1,896
2019 + 2021 LS Means	2	S2_10777410	0.053	−	GRMZM2G068982	5,033

^*^ Chromosome.

^^^ Single Nucleotide Polymorphism.

The significant SNPs identified in the 2019 + 2021 combined dataset and for incubation period are shown.

Resistance to diseased leaf area (DLA) is genetically distinct from incubation period; a single marker was significant on chromosome 2 (**Figure 3C**). The significant marker, S2_10777410 (*p =* 0.053), is proximal to GRMZM2G068982, a methionine aminopeptidase. Using the same marker dataset, a stepwise regression approach did not reveal any significant markers for the field DLA dataset (**Table 5**). Stepwise regression is more conservative than GLM, and our findings are consistent in that no significant markers were identified using stepwise regression.

There was no overlap in significant markers for incubation period and disease severity. We observed a weak negative correlation between disease severity and incubation period (**Table 3**). Although we observed some chlorotic resistant responses in our 2019 and 2021 field experiments and 2020 greenhouse experiment, we did not identify any significant markers near any of the major genes for NCLB resistance, *Ht1, Ht2, Ht3, HtN1* (**Figure S1**). If the qualitative resistance genes were present in the population, they may have been at too low of a frequency to detect any significant markers in these regions. It is also possible that these responses were due to novel resistance genes.

### 3.3 Metabolic pathways associated with disease resistance

To better understand the molecular mechanisms associated with resistance to NCLB we conducted a metabolic pathway analysis and examined a previously published RNA-seq dataset. We identified metabolic pathways associated with disease severity and incubation period using the results from the GLM model and the PAST software (Thrash et al., 2020). In total, 24 metabolic pathways (α = 0.05) were associated with resistance to *E. turcicum* (**Table 6**), including nine associated with incubation period and 15 associated with disease severity. Several pathways associated with plant hormones were significant including jasmonic acid biosynthesis (PWY-735, *p* = 0.02) and cytokine-O-glucosides biosynthesis (PWY-2902, *p* = 0.04) in the combined disease severity dataset. Additionally, gibberellin inactivation I (PWY-102, *p =* 0.01) and brassinosteroids inactivation (PWY-6546, *p =* 0.03) were significant in the incubation period dataset. A pathway of particular interest is the ethylene biosynthesis pathway (ETHYL-PWY) which was significant for both incubation period (*p =* 0.006) and disease severity (*p =* 0.01). Ethylene synthesis has previously been associated with disease resistance in plants (Dong, 1987; van Loon et al., 2006). Additionally, S-adenosyl-L-methionine cycle II (PWY-5041) was significant for disease severity. S-adenosyl-L-methionine (SAM) is the starting substrate in ethylene synthesis. It is converted to 1-aminocyclopropane-1-carboxylic acid (ACC) by ACC synthase (ACS), which is subsequently converted into ethylene via oxidation by ACC oxidase (ACO) (Xu and Zhang, 2015).

**Table 6:** Significant metabolic pathways associated with increasing or decreasing incubation period and the 2019 + 2021 combined disease severity datasets.

Pathway ID	Pathway Name	*p*-Value	Effect	Dataset
ETHYL-PWY	ethylene biosynthesis I (plants)	0.0062419	Decrease	IP^*^
PWY-5667	CDP-diacylglycerol biosynthesis I	0.0273528	Decrease	IP
PWY-5138	unsaturated, even numbered fatty acid-oxidation	0.0354694	Decrease	IP
PWY-3181	tryptophan degradation VI (via tryptamine)	0.0367993	Decrease	IP
PWY-581	indole-3-acetate biosynthesis II	0.0459157	Decrease	IP
PWY-5080	very long chain fatty acid biosynthesis I	0.0026768	Increase	IP
PWY-102	gibberellin inactivation I (2-hydroxylation)	0.0152976	Increase	IP
PWY-5097	lysine biosynthesis VI	0.0268191	Increase	IP
PWY-6546	brassinosteroids inactivation	0.0334693	Increase	IP
LIPASYN-PWY	phospholipases	0.007419	Decrease	DLA^#^
PWY-6441	spermine and spermidine degradation III	0.011873	Decrease	DLA
PWY-735	jasmonic acid biosynthesis	0.022039	Decrease	DLA
PWY-5097	lysine biosynthesis VI	0.032238	Decrease	DLA
PWY-6959	L-ascorbate degradation V	0.032430	Decrease	DLA
PWY-6803	phosphatidylcholine acyl editing	0.037275	Decrease	DLA
PWY-2902	cytokinin-O-glucosides biosynthesis	0.048633	Decrease	DLA
PWY-5041	S-adenosyl-L-methionine cycle II	0.007802	Increase	DLA
ETHYL-PWY	ethylene biosynthesis I (plants)	0.014177	Increase	DLA
PWY-5690	TCA cycle II (plants and fungi)	0.019701	Increase	DLA
PWY-6363	D-myo-inositol (1,4,5)-trisphosphate degradation	0.021242	Increase	DLA
PWY-702	methionine biosynthesis II	0.026966	Increase	DLA
PWY-6549	glutamine biosynthesis III	0.031528	Increase	DLA
UDPNACETYLGALSYN-PWY	UDP-N-acetyl-D-glucosamine biosynthesis II	0.047356	Increase	DLA
PWY-5138	unsaturated, even numbered fatty acid-oxidation	0.047695	Increase	DLA

^*^IP, incubation period.

#DLA, diseased leaf area.

Only pathways significant at α *=* 0.05 are shown.

Pathogens are known to mimic phytohormones and interfere with signaling (Denance et al., 2013), as well as to target regulatory components involved with *in planta* hormone production (Robert-Seilaniantz et al., 2011). Thus, we examined an RNA-seq dataset (Adhikari et al., 2021) for DEGs associated with ethylene biosynthesis during the early stages of infection. We examined three contrasts: inoculated versus uninoculated maize 24 hai, inoculated versus uninoculated maize 72 hai, and inoculated maize 24 versus 72 hai. Only one gene associated with ethylene biosynthesis, GRMZM2G07529 (FDR = 0.03), was upregulated, with a fold change of 16.87, in maize 24 hai with *E. turcicum* compared to maize 24 hai with DI H_2_O. GRMZM2G07529 is annotated as ACC oxidase *ACO31*. ACO is the final oxidation step in the synthesis of ethylene (Xu and Zhang, 2015). Thus, during the initial phases of infection ethylene production may be increased, indicating that it may play a role in resistance to NCLB.

### 3.4 Investigation of ethylene and resistance to *E. turcicum*

We hypothesized that inhibiting ethylene would alter NCLB disease severity. We used a foliar treatment of AgNO_3_ to examine the effect of ethylene on disease resistance. AgNO_3_ inhibits ethylene action *in planta* by reducing the ability of ethylene receptors to bind to ethylene, which results in decreased ethylene sensitivity and the inhibition of continuous ethylene production (Kumar et al., 2009). We hypothesized that inhibition of ethylene by applying AgNO_3_ would decrease disease severity and increase incubation period. Furthermore, we postulated that host resistant level and genetic background might alter the magnitude by which inhibiting ethylene improves resistance and increases incubation period.

**Figure 4:**
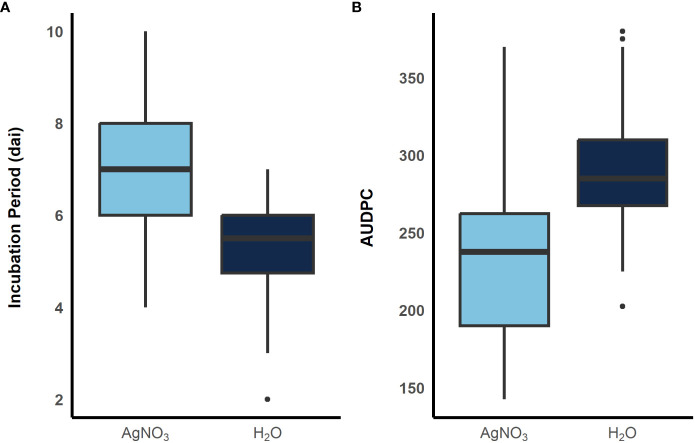
Boxplots showing distribution of incubation period **(A)** and AUDPC **(B)** when plants were sprayed with AgNO_3,_ light blue, and sterile water, dark blue, before inoculation with *E. turcicum.* A total of 12 plants were replicated three times in each treatment. (300 x 115 mm, 300 dpi).

To examine the role of ethylene in *E. turcicum* infection, a total of 10 DH lines and both recurrent parents were selected. We selected DH lines in pairs within each exotic family, where the pair had contrasting disease phenotypes. Plants were either treated with AgNO_3_ or DI H_2_O prior to a whorl inoculation with *E. turcicum*. From our ethylene inhibition assay, disease severity was significantly impacted by genotype (**Table 7**, *p* = 0.012) and by treatment with AgNO_3_ or deionized H_2_O (**Table 7**, *p* = 1.756 × 10^-5^). Similarly, incubation period was significantly impacted by genotype (**Table 8**, *p* = 0.004) and by treatment with AgNO_3_ or deionized H_2_O **Table 8**, *p* = 4.281 × 10^-5^). In plants treated with AgNO_3_, incubation period increased and AUDPC decreased (**Figure 4**). We did not observe a significant impact of the line’s resistance level, exotic family, or recurrent parent on AUDPC or incubation period (α = 0.05). In this experiment, application of AgNO_3_ affected all genotypes similarly meaning that, of the lines screened, no line was more or less resistant relative to the other lines after AgNO_3_ application; thus, inhibiting ethylene using AgNO_3_ decreases disease severity similarly in the lines screened.

**Table 7:** Analysis of variance (ANOVA) output from the model used to evaluate the split-plot ethylene design to assess the effect of whole-plot and subplot on AUDPC.

Source	DF^*^	SS^#^	MS^^^	F-value	P-value
Whole Plot	2	5524	2762.1	1.6573	0.20764
Genotype	11	51549	4686.3	2.8117	0.01209
Whole Plot/Treatment	3	62622	20873.9	12.5241	1.756 × 10^-5^
Whole Plot × Genotype	22	35546	1615.7	0.9694	0.52271
Error	30	50001	1666.7		

^*^DF, degrees of freedom.

#SS, sums of squares.

^^^MS, mean squares.

**Table 8:** Analysis of variance (ANOVA) output from the model used to evaluate the split-plot ethylene design to assess the effect of whole-plot and subplot on incubation period.

Source	DF^*^	SS^#^	MS^^^	F-value	P-value
Whole Plot	2	0.75	0.375	0.3123	0.7339
Genotype	11	43.042	3.9129	3.2587	0.00417
Whole Plot/Treatment	3	38.875	12.9583	10.7918	4.281 × 10^-5^
Whole Plot × Genotype	22	30.583	1.3902	1.1577	0.34438
Error	33	39.625	1.2008		

^*^DF, degrees of freedom.

#SS, sums of squares.

^^^MS, mean squares.

## 4 Discussion

We evaluated the exotic-derived BGEM population for resistance to NCLB and examined the role of metabolic pathways in resistance to NCLB, specifically ethylene. We identified several significant markers for diseased leaf area and incubation period. These markers can be used to enrich the repertoire of quantitative resistance genes effective against *E. turcicum*. Additionally, we report that the ethylene synthesis pathway modulates a defense response against *E. turcicum*. Finally, we report variation in the ethylene metabolic pathway as a whole that is associated with resistance to NCLB.

This is the first study to report on disease resistance in the BGEM population. While we did not map any previously identified qualitative genes, several interesting responses were observed during the early stages of infections in some lines (**Figure S1**). Of note, the lines BGEM-0081-S, BGEM-0027-S, BGEM-0087-S, BGEM-0116-S, and BGEM-0154-S showed defense responses that were the most unique, could be useful in breeding for resistance and are worth examining closer in future experiments (**Figure S1**). Allelic variation has been reported for the major genes *Ht2/3* (Hurni et al., 2015; Yang et al., 2021), and it would be reasonable to expect allelic variation for other qualitative resistance genes.

We identified three markers associated with incubation period and one marker associated with DLA (**Table 5**). We observed no overlap between the significant markers associated with incubation period and the marker associated with diseased leaf area indicating that the genetic architecture underlying disease severity and incubation period are distinct. It was surprising to see no overlap in significant markers for disease severity and incubation period; however, this phenomenon has been reported previously (Balint-Kurti et al., 2010). Incubation period and disease severity are important measures of resistance to *E. turccium.* Both traits are typically strongly correlated (Smith and Kinsey, 1993; Welz and Geiger, 2000). In this study we observed significant, weak negative correlations between disease severity and incubation period (**Table 3**), suggesting that the ability to delay the onset of lesions is a separate component of resistance than restricting the pathogen growth after initial lesion development. Both incubation period and disease severity are believed to be controlled by genetically similar mechanisms (Schechert et al., 1999; Welz et al., 1999).

Of the three significant markers associated with incubation period, one marker (S3_8266879) was significant after the stepwise regression approach in TASSEL and is within GRMZM2G108898, a plant cysteine oxidase 1 (PCO1). Plant cysteine oxidases are associated with oxygen sensing and stress response in plants (Weits et al., 2014). When under hypoxic conditions, such as flooding, PCOs initiate downstream ethylene production by stabilizing ethylene response factors (ERFs) such as ERF-VII (Hartman et al., 2021). In Arabidopsis, several PCOs were found to be sensitive to *in planta* oxygen levels and necessary for mediating the stability of ERF-VII transcription factors in low oxygen environments (White et al., 2018). Because ethylene is a stress hormone, it makes sense that ERFs would be important in plant hosts under abiotic and biotic stress factors. In maize two ERFs, *ZmERF061* and *ZmERF105*, have been implicated as important for NCLB resistance (Zang et al., 2020; Zang et al., 2021).

We identified a single marker associated with disease severity, S2_10777410. This marker is proximal to a methionine aminopeptidase, GRMZM2G068982. Methionine aminopeptidases function as catalysts in cleaving methionine from synthesized polypeptides. Methionine is involved in many metabolic pathways, including the production of ethylene. The ethylene biosynthesis pathway was significant (α = 0.05) in the post-GWAS PAST analysis. Interestingly, *ACO* was strongly upregulated in maize plants 24 hai with *E. turcicum* compared to mock inoculated control. It is known that ethylene production is upregulated in response to pathogen invasion (Li et al., 2012; Xu and Zhang, 2015). The final step in ethylene synthesis is the oxidation of ACC by ACO which yield ethylene. The upregulation of *ACO* during the initial stages of infection supports our hypothesis that ethylene is an important defense-related hormone for resistance against NCLB. In our ethylene experiment, we saw that the inhibition of ethylene significantly increased incubation period and significantly decreased disease severity. This same trend has been observed in other pathosystems, where inhibiting ethylene or decreasing ethylene production improves resistance (Dong, 1987; van Loon et al., 2006; Wang et al., 2017).

The role of ethylene in plant defense against biotic stress factors, such as pests and pathogens, is highly complex, as both the synthesis and response to ethylene can be modulated to confer resistance or susceptibility. In some pathosystems it has also been shown that plant sensitivity to ethylene production is important in mediating resistance. Ethylene insensitive mutants in Arabidopsis had higher disease severity to necrotrophic and hemibiotrophic pathogens, while disease severity was lower for biotrophic pathogens (van Loon et al., 2006). In rice, increased accumulation of ethylene increased host susceptibility to rice dwarf virus (Zhao et al., 2017), but decreased ethylene accumulation resulted in decreased resistance to the hemibiotrophic pathogen *Magnaporthe oryzae* (Zhai et al., 2022). In tomato, plants with antisense mutations for the ERF *LeETR4* were generally more resistant to the bacterial pathogen *Xanthomonas campestris* pv. *vesicatoria*, suggesting expression of ERFs may be important in rapid response to pathogen invasion (Ciardi et al., 2001). Two ERFs have been identified in maize. Transcription of these ERFs are upregulated during *E. turcicum* infection, suggesting that ethylene sensitivity may be as important as ethylene production in defense response (Zang et al., 2020; Zang et al., 2021). Given the complexity of the role of ethylene in stress response, it is likely that there are many pleiotropic effects associated with ethylene synthesis and sensing.

After ethylene is synthesized, there are several downstream effects, including interactions with JA, as well as potential pleiotropic roles. In Arabidopsis it was shown that ethylene biosynthesis is activated due to insect herbivory, with downstream effects, such as increased transcription of ethylene response factors and JA synthesis (Rehrig et al., 2014). Additionally, in Arabidopsis a calcium elongation factor and a glutathione S-transferase were upregulated when ethylene production was increased (Stotz et al., 2000). Other downstream effects of ethylene synthesis include increased callose accumulation and increased expression of *Tdy2*, enhancing overall resistance to aphids in maize (Varsani et al., 2019). In maize, ethylene production is shown to mediate the expression of *mir-1*, an insect defense related gene, and is believed to also interact with JA (Harfouche et al., 2006). Metabolite abundance has been implicated and ethylene synthesis have been shown to be interconnected in mediating resistance to the necrotrophic pathogen *Fusarium graminearum* (Zhou et al., 2019).

Most research has investigated the qualitative role of ethylene in plant-pathogen interactions. However, little is known about the effect of allelic variation in ethylene related genes on disease severity. Here we show that there is variation in the ethylene metabolic pathway as a whole that is signiciantly associated with NCLB resistance. Our study is limited in that we did not measure ethylene production or the expression of genes associated with the production or response to ethylene. However, the ethylene inhibition experiment suggests that ethylene is an important part of the maize-*E. turcicum* pathosystem. Other areas of exploration would be to measure quantitative differences in ethylene production in response to pathogen inoculation in lines with allelic diversity in the ethylene pathway, as well as to evaulate mutants and natural alleles of genes related to ethylene biosynthesis and sensing. Additionally, future studies could examine the effect of the exogenous application of ethylene on gene expression and disease resistance. While no individual genotypes were statistically different between treatment with AgNO_3_ and sterile water, we observed a range in disease severity in each treatment. Based on our findings, it is plausible that allelic variation in genes associated with ethylene production and ethylene sensitivity could translate to altered defense responses.

## 5 Conclusion

Northern corn leaf blight is an economically important disease. We screened the BGEM population for resistance to NCLB, identified metabolic pathways associated with resistance to NCLB, and confirmed the role of ethylene in resistance to NCLB. In this study we demonstrate the utility of the BGEM population as a source of novel alleles for resistance, and contribute to the growing body of literature focused on the role of ethylene in plant-microbe interactions, specifically maize-*E. turcicum.* In this study, we contribute to the growing body of literature by examining the role of ethylene in NCLB resistance.

## Data availability statement

The datasets presented in this study can be found in online repositories. The names of the repository/repositories and accession number(s) can be found in the article/**Supplementary material**.

## Author contributions

SL: Conceptualization, Formal Analysis, Investigation, Methodology, Validation, Visualization, Writing – original draft. AL: Methodology, Validation, Writing – review and editing. SM: Conceptualization, Methodology, Resources, Writing – review and editing. TJ: Conceptualization, Data curation, Funding acquisition, Methodology, Project administration, Resources, Supervision, Writing – review and editing.

## Supplementary material

Supplementary Figure 1 Variation in chlorotic responses during the early stages of *E. turcicum* infection and colonization in multiple BGEM lines, **(A)** BGEM-0081-S, **(B)** BGEM-0027-S, **(C)** BGEM-0087-S, **(D)** BGEM-0016-S, **(E)** BGEM-0154-S.
