# Supplementary material for: Inhibition of ethylene involved in resistance to E. turcicum in an exotic-derived double haploid maize population
Source: Front Plant Sci. 2023 Oct 6;14:1272951. doi: 10.3389/fpls.2023.1272951 (PMC10587583; doi:10.3389/fpls.2023.1272951)
Supplement: Supplementary Figure 1 — Variation in chlorotic responses during the early stages of E. turcicum infection and colonization in multiple BGEM lines, (A) BGEM-0081-S, (B) BGEM-0027-S, (C) BGEM-0087-S, (D) BGEM-0016-S, (E) BGEM-0154-S. [file Image_1.pdf]

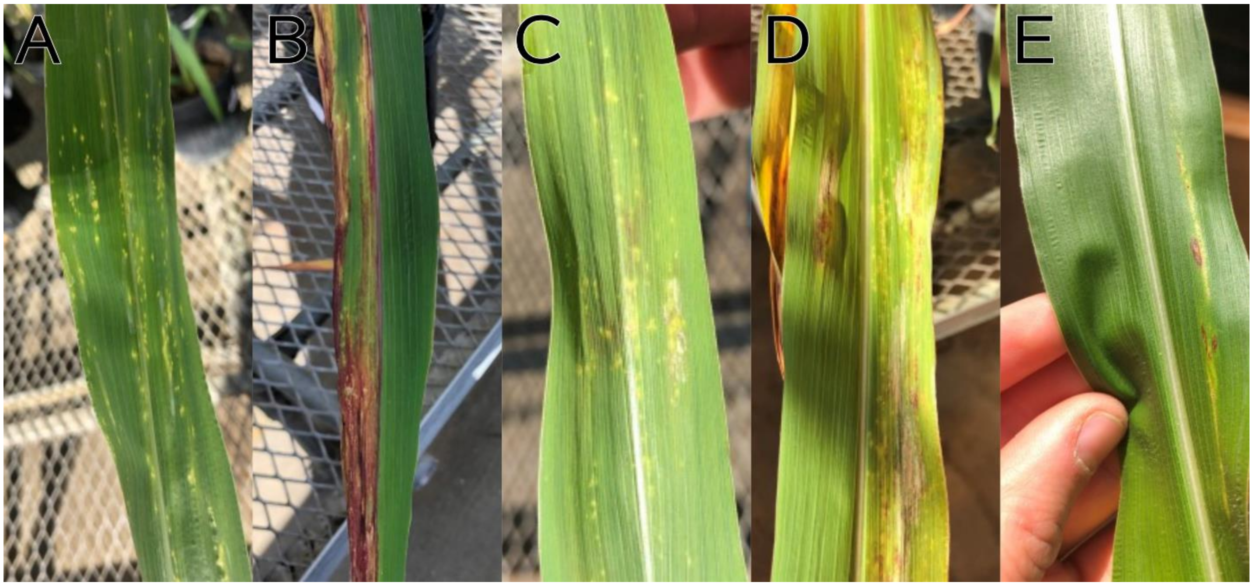

Supplementary Figure S1. Variation in chlorotic responses during the early stages of E. turcicum infection and colonization in multiple BGEM lines, **A)** BGEM-0081-S, **B)** BGEM-0027-S, **C)** BGEM-0087-S, **D)** BGEM-0016-S, **E)** BGEM-0154-S.
